# Functionalized Surface Geometries Induce: “*Bone: Formation by Autoinduction”*

**DOI:** 10.3389/fphys.2017.01084

**Published:** 2018-02-06

**Authors:** Ugo Ripamonti

**Affiliations:** Bone Research Laboratory, Faculty of Health Sciences, School of Oral Health Sciences, University of the Witwatersrand, Johannesburg, South Africa

**Keywords:** geometry, nanopatterned surface topography, geometric induction of bone formation, stem cells and differentiation, intrinsic induction of bone formation, bone morphogenetic proteins

## Abstract

The induction of tissue formation, and the allied disciplines of tissue engineering and regenerative medicine, have flooded the twenty-first century tissue biology scenario and morphed into high expectations of a fulfilling regenerative dream of molecularly generated tissues and organs in assembling human tissue factories. The grand conceptualization of deploying soluble molecular signals, first defined by Turing as forms generating substances, or morphogens, stemmed from classic last century studies that hypothesized the presence of morphogens in several mineralized and non-mineralized mammalian matrices. The realization of morphogens within mammalian matrices devised dissociative extractions and chromatographic procedures to isolate, purify, and finally reconstitute the cloned morphogens, found to be members of the transforming growth factor-β (TGF-β) supergene family, with insoluble signals or substrata to induce *de novo* tissue induction and morphogenesis. Can we however construct macroporous bioreactors *per se* capable of inducing bone formation even without the exogenous applications of the osteogenic soluble molecular signals of the TGF-β supergene family? This review describes original research on coral-derived calcium phosphate-based macroporous constructs showing that the formation of bone is independent of the exogenous application of the osteogenic soluble signals of the TGF-β supergene family. Such signals are the molecular bases of the induction of bone formation. The aim of this review is to primarily describe today's hottest topic of biomaterials' science, i.e., to construct and define osteogenetic biomaterials' surfaces that *per se*, in its own right, do initiate the induction of bone formation. Biomaterials are often used to reconstruct osseous defects particularly in the craniofacial skeleton. Edentulism did spring titanium implants as tooth replacement strategies. No were else that titanium surfaces require functionalized geometric nanotopographic cues to set into motion osteogenesis independently of the exogenous application of the osteogenic soluble molecular signals. Inductive morphogenetic surfaces are the way ahead of biomaterials' science: the *connubium* of stem cells on primed functionalized surfaces precisely regulates gene expression and the induction of the osteogenic phenotype.

## The new frontiers in bone tissue engineering: inductive biomimetic functionalized surfaces beyond morphogens and stem cells

We have often stated that “*bone tissue engineering starts by erecting scaffolds of biomimetic matrices controlling the expression of the soluble molecular signals of the transforming growth factor-*β *(TGF-*β*) supergene family*” (Ripamonti, 2006; Ripamonti et al., 2007a). We have also stated that the “*future of the continuous evolution of biomaterials is to functionalize the implanted biomaterials surfaces by activating the surface biology to directly induce specific molecular and tissue biology phenomena initiating regenerative responses as inductive biomaterials*” (Ripamonti et al., 1993; Ripamonti, 2009; Klar et al., 2013).

Regenerative medicine and tissue engineering are the grand multidisciplinary challenges of “*the science of fabricating new tissues for replacement and total regeneration*” (Reddi, 1994). Moreover, the challenges “*of design and manufacture of new tissues for the functional restoration of the impaired organs and replacement of lost parts*” of the human body (Reddi, 2000). The ultimate challenge of regenerative medicine and tissue engineering is, however, to explore *de novo* and *ex novo* tissue induction and morphogenesis (Reddi, 1997, 2000) hypothesized in human tissue factories, firstly in academic research laboratories, and possibly later in biotechnology companies.

The isolation, purification and later the molecular cloning of the osteogenic proteins of the TGF-β supergene family (Ripamonti, 2003, 2006) did finally result in the widespread pre-clinical application of the “*bone induction principle*” (Urist et al., 1967) with translation in clinical context of the available recombinant human proteins (Wozney et al., 1988; Reddi, 2000; Ripamonti et al., 2004; Ripamonti, 2006). Recombining soluble signals with insoluble signals or substrata defined the basic tissue engineering paradigm (Sampath and Reddi, 1981, 1983; Reddi, 1997, 2000). The mechanical reconstitution of both soluble and insoluble signals initiate the induction of bone formation (Sampath and Reddi, 1981, 1983; Ripamonti et al., 1992, 2000; Reddi, 1994, 1997, 2000; Ripamonti, 2006). Continuous experimentation in preclinical contexts in primate models including the Chacma baboon *Papio ursinus* has indicated that there is no induction of bone in absence of the osteogenic proteins of the TGF-β supergene family (Ripamonti, 2003, 2005).

The emerging question and the novel frontiers of biomaterials' science is whether biomaterial scientists and developmental molecular biologists alike can assemble self-initiating biomimetic matrices that *per se* set into motion “*Bone: formation by autoinduction*” (Urist, 1965). Importantly, the induction of bone initiates without exogenously applied soluble osteogenic molecular signals of the TGF-β supergene family, powerful initiators of tissue patterning and morphogenesis (Wozney et al., 1988; Ripamonti et al., 1992; Reddi, 1997, 2000; Ripamonti, 2003, 2006). Can we engineer calcium phosphate-based biomimetic matrices whereby differentiating myoblastic/myoendothelial and/or pericytic/perivascular/endothelial stem cells will express osteogenic mRNA species of the TGF-β family? Secreted gene products will then initiate the morphogenesis of bone as a secondary response (Ripamonti et al., 1993, 1999).

Within the context of biomimetism, biomimetic matrices and the induction of bone formation (Ripamonti, 2009; Ripamonti and Roden, 2010), newly designed biomimetic matrices have set a new lexicon of regenerative medicine and tissue engineering. The lexicon has been enriched by novel terms, such as biomimetics, biomimetism so as to biomimetizes the complex functional structural multi-million years-old tested extracellular matrix topographies, designs, and geometries of animal phyla to invocate the “*creative initiation of various specific biological systems gaining inspiration from Nature*” (Sarikaya, 1999; Ripamonti, 2009; Williams, 2014).

This spontaneous and/or intrinsic osteoinductivity (Ripamonti, 1996) has defined an additional tissue engineering paradigm whereby biomimetic matrices *per se* spontaneously initiate the induction of bone formation within the macroporous spaces of certain calcium phosphate-based bioreactors (Ripamonti, 1990, 1991, 1996, 2004, 2006; Ripamonti et al., 1993, 1999; van Eeden and Ripamonti, 1994).

**Figure 1:**
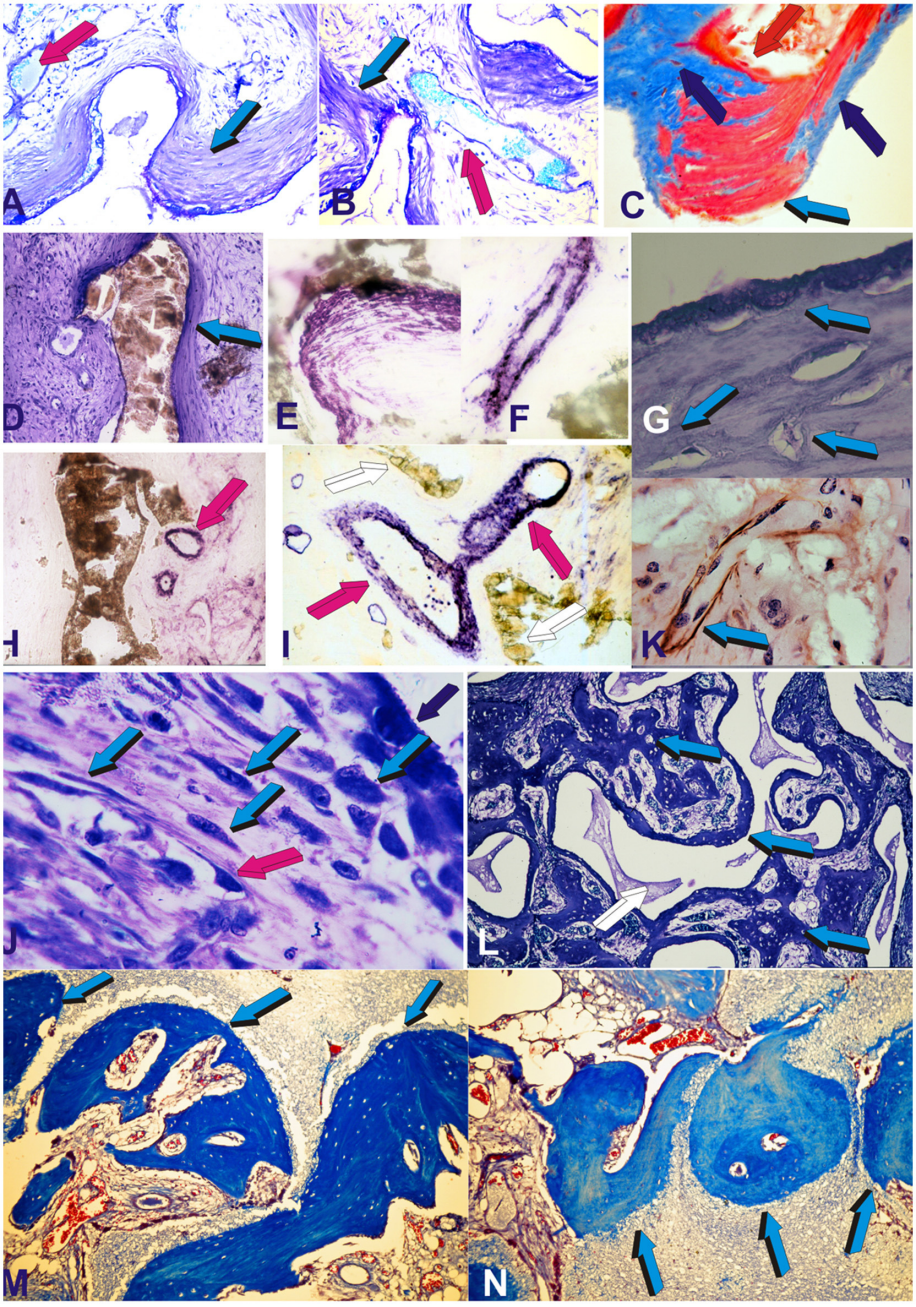
Self-inducing geometric cues and the induction of tissue patterning, morphogenesis with the final induction of bone formation as initiated by the geometric concavities of the coral-derived calcium phosphate based macroporous bioreactors. Coral-derived macroporous bioreactors, 20 mm in height and 11 mm in diameter, were implanted in heterotopic intramuscular *rectus abdominis* sites in a series of non-human primate Chacma baboon *Papio ursinus*. Generated tissues were harvested on day 30, 60, and 90 after heterotopic implantation (Ripamonti, 1990, 1991, 1996; Ripamonti et al., 1993, 2009, 2010; van Eeden and Ripamonti, 1994). Harvested tissues were processed for decalcified and undecalcified histological analyses. **(A–D)** Tissue patterning and the induction of mesenchymal tissue condensations on day 30 (day 90 in **C**) at the hydroxyapatite interface (light blue arrows) with capillary invasion (magenta arrows). Alkaline phosphatase staining and activity within both mesenchymal condensations (on day 60) **(E)** and capillary sprouting and invasion (on day 30) **(F)**. **(G)** Patterning and further morphogenesis of collagenous condensations at the hydroxyapatite interface with the development of osteoblast-like cells within the differentiating and remodeling condensations on day 30 (light blue arrows). **(H,I)** Further alkaline phosphatase staining (magenta arrows) of invading capillaries on day 30. Alkaline phosphate stains intensely within the multiple cellular layers of the sprouting branching capillaries in close contact with the hydroxyapatite substratum. **(K)** Macroporous construct harvested on day 60 shows laminin immunolocalization (light blue arrow) within invading capillaries penetrating the macroporous spaces. Type IV collagen and laminin' amino acid motifs bind both angiogenic and bone morphogenetic proteins sequences which may be released during tissue induction and morphogenesis to initiate the induction of bone formation (Ripamonti, 2006, 2010 for reviews) Note the intimacy of laminin immunolocalization with large hyperchromatic endothelial cells, possibly preparing to migrate out of the vascular compartment. **(J)** On day 60, there is a continuous flow of responding mesenchymal cells (light blue arrows) moving from the vascular Trueta's angiogenic vessels (magenta arrow) to the osteoblastic/osteogenetic differentiation site (dark blue arrow). The digital image in **(J)** depicts the critical differentiating role of the nanopatterned geometric substratum on the induction of cellular differentiation, osteoblast synthesis and the induction of bone formation directly attached to the self-inducing nanopatterned calcium phosphate-based macroporous bioreactor (Ripamonti et al., 1993). **(L)** Bone morphogenesis later develops on day 90 within the macroporous spaces of the coral-derived bioreactors, with woven bone (light blue arrows in **L**) with woven bone initiating within concavities of the substratum. **(M,N)** Bone induction and remodeling of the newly formed bone within concavities (light blue arrows) on day 90 after *rectus abdominis* implantation. Decalcified and undecalcified paraffin wax and Historesin sections cut at 3 to 6 μm and stained with toluidine blue in 70% ethanol or **(C)** stained free-floating with modified Goldner's trichrome stain **(C)**. Decalcified sections **(M,N)** also stained with modified Goldner's trichrome stain.

In early experiments in the Chacma baboon *Papio ursinus* (Ripamonti, 1990, 1991) heterotopically implanted coral-derived macroporous bioreactors within the *rectus abdominis* muscle, unexpectedly showed the morphogenesis of bone within the macroporous spaces of the implanted bioreactors by day 90 (Ripamonti, 1990, 1991; Figure 1). These were the first experiments that unambiguously showed the morphogenesis of bone in macroporous calcium phosphate-based biomaterials after heterotopic intramuscular implantation and without the addition of the osteogenic proteins of the TGF-β supergene family (Ripamonti, 1990, 1991).

**Figure 2:**
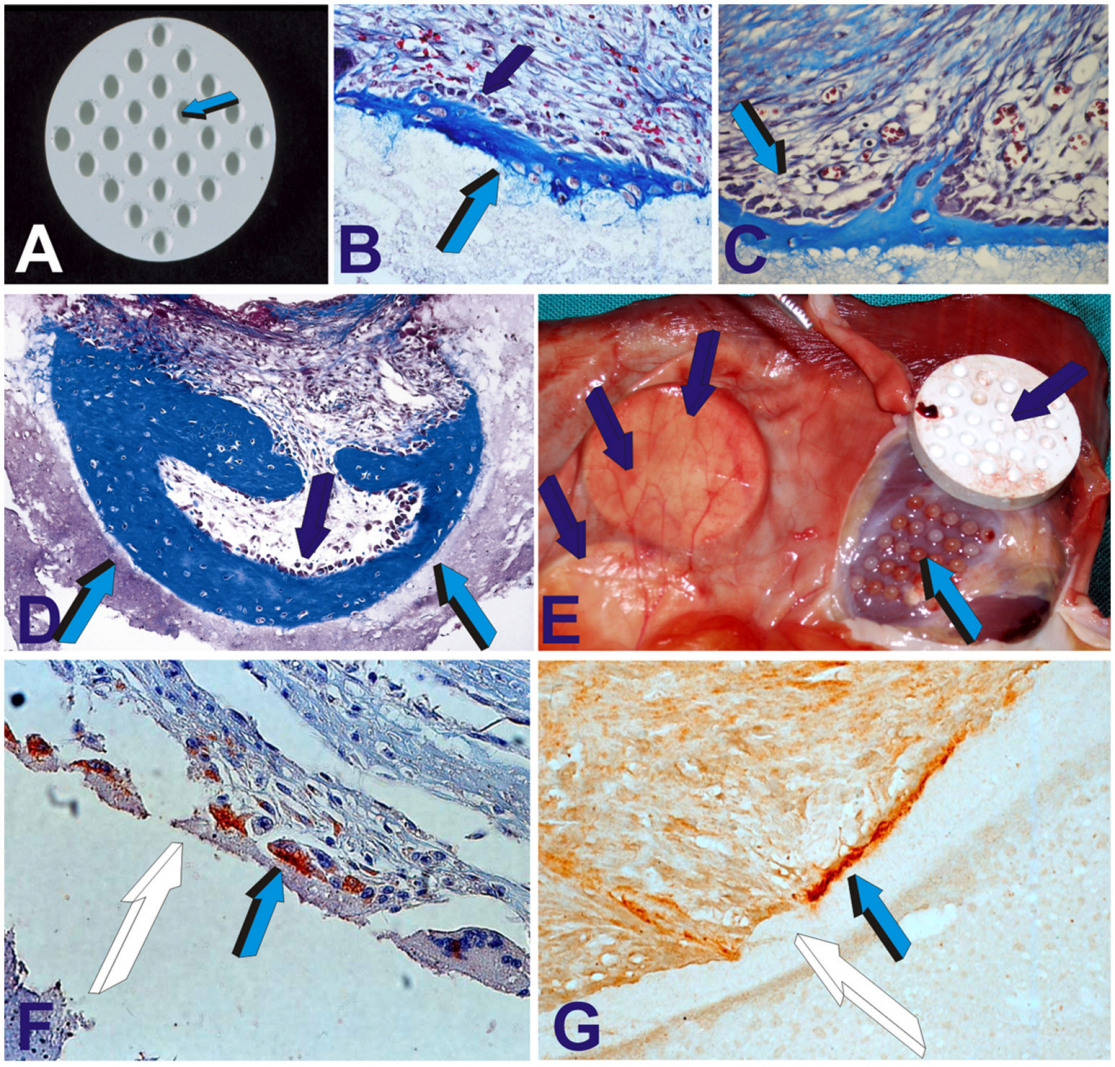
The geometric induction of bone formation: the concavity, the shape of life. Concavities (Ripamonti et al., 1999; Ripamonti, 2006), as prepared in solid discs of highly crystalline sintered hydroxyapatite constructs on both planar surfaces (Ripamonti et al., 1999), **(A)** spontaneously initiate the induction of bone formation on day 30 after *rectus abdominis* implantation **(B,C)**. The newly formed intramembranous bone is tightly attached to the hydroxyapatite biomatrix (**B** light blue arrow) anchoring into the hydroxyapatite substratum **(B,C)**. On day 90 **(D)**,the newly formed bone within the concavity (light blue arrows) remodels still surfaced by contiguous osteoblasts (dark blue arrow) continuously secreting bone matrix. **(E)** Tissue harvest of highly crystalline sintered constructs (dark blue arrows) implanted in the *rectus abdominis* muscle. Highly vascularized mesenchymal tissue (light blue arrow) had grown within the concavities of the harvested sintered construct (dark blue arrow). **(F)** On day 30, differentiating cells at the hydroxyapatite interface express bone morphogenetic protein-7 (also known as osteogenic protein-1, OP-1) within the cytoplasm (light blue arrow) later embedded as gene product **(G)** (light blue arrow) within the microstructure of the hydroxyapatite biomatrix (white arrows).

Our systematic experimentation in *Papio ursinus* using coral-derived macroporous constructs extensively studied morphologically (Ripamonti, 1990, 1991, 2009; Ripamonti et al., 1993) and immuno-histochemically (Ripamonti et al., 1993) and by Northern blot analyses (Ripamonti et al., 2009), the induction of bone formation as initiated by coral-derived bioreactors (Figures 1, 2), additionally investigating the heterotopic microenvironment of the *rectus abdominis* muscle in different animal models (Ripamonti, 1996).

Multiple and systematic experiments in *Papio ursinus* showed that the differentiation of compact mesenchymal condensations against the coral-derived surface was one of the earliest morphogenetic event predating the induction of bone formation within the macroporous spaces (Ripamonti, 1990, 1991; Figure 1). Together with differentiating mesenchymal condensations at the hydroxyapatite interface there is always angiogenesis and capillary sprouting within the macroporous spaces intimately associated with mesenchymal condensations (Figures 1B–J; Ripamonti et al., 1993). Angiogenesis provides perivascular pericytic cells for the differentiation of the osteogenic phenotype (Figures 1F,H,I). Of interest, the induction of mesenchymal tissue condensations predating the formation of bone in coral-derived macroporous bioreactors, replicates and/or biomimetizes the induction of condensations that characterize the induction of intramembranous bone formation.

Morphologically and biochemically, time course experiments showed a temporally and spatially correlated expression' sequence of alkaline phosphatase which was found to be intimately localized to the invading vasculature (Figures 1F,H,I) and subsequently to mesenchymal condensations at the hydroxyapatite interface (Figures 1D,E; Ripamonti et al., 1993). Of note, immuno-histochemical studies showed that the newly formed capillaries invading the macroporous spaces were positive for alkaline phosphatase expression and were intimately associated with developing osteoblasts (Figures 1F,H,I). The basement membrane of the forming capillaries generating within the macroporous spaces showed laminin expression by day 60 after heterotopic implantation (Figure 1K). Invading capillaries are thus the osteogenetic vessels of Trueta' definition that pattern and define the induction of bone formation in angiogenesis (Trueta, 1963; Ripamonti, 2006; Ripamonti et al., 2007a).

The temporal sequence of alkaline phosphatase expression firstly within invading capillaries on day 30 (Figures 1F,H) and later within collagenous condensations at the hydroxyapatite interface (Figure 1E) indicate a spatio/temporal flow of invading activated precursor cells available from the osteogenetic vessels (Figures 1F,I,J; Trueta, 1963) for later differentiation at the hydroxyapatite interface (Figure 1J).

Critical experiments have shown the binding of both morphogenetic and angiogenic proteins to the extracellular matrix of the basement membrane components of the invading capillaries including laminin and type IV collagen (Vlodavsky et al., 1987; Folkman et al., 1988; Paralkar et al., 1990, 1991). Binding and incorporation of both morphogenetic and angiogenetic proteins to selected basement membrane components elevate the osteogenetic capillaries to morphogenetic inductive capillaries of Aristotle' definition (Ripamonti, 2010).

We did state in previous published work that the binding and sequestration of both angiogenetic and osteogenic proteins provide the “*conceptual framework of the supramolecular assembly of the extracellular matrix of bone*” (Ripamonti, 2006, 2010; Ripamonti et al., 2007a). Angiogenetic and osteogenetic proteins have been shown to bind to Type IV collagen (Vlodavsky et al., 1987; Folkman et al., 1988; Paralkar et al., 1990, 1991). Type IV collagen is part of the extracellular matrix' basement membrane of the invading capillaries, and bound morphogenetic and angiogenetic proteins are thus presented in an immobilized form to responding mesenchymal cells (Paralkar et al., 1990, 1991; Ripamonti, 2006). The molecular arrangement of the sequestered proteins, together with the molecular and anatomical stemness within perivascular stem cell niches originating from both the perivascular pericytes and/or myoblastic cells including myoendothelial cells of the striated *rectus abdominis* muscle, set the supramolecular assembly of the spontaneous induction of bone formation within the striated *rectus abdominis* primate' muscle (Ripamonti et al., 2007a; Ripamonti, 2009, 2010).

There is thus a complex yet fine tuning of molecular and cellular cross-talk between invading capillaries and morphogenetic soluble signals. Morphogens have been shown to be attached to selected extracellular matrix components of the capillaries, including specific amino-acid motifs of laminin within the capillaries' extracellular matrix, and responding cells, primarily differentiating whilst stretching on nanotopographic geometric configurations. It has been proposed that differentiating osteoblast-like cells may “*see*” laminin' selected amino-acid sequence' motifs (Vukicevic et al., 1990) setting into motion the ripple-like cascade of the induction of bone formation (Ripamonti, 2006, 2010; Ripamonti et al., 2007a).

Figure 1K shows the exquisite and intimate relationships of laminin immunolocalization within the basement membrane of an invading capillary with lining hyperchromatic endothelial cells possibly preparing to migrate out of the vascular compartment. The molecular deduction is morphologically sustained by the exquisite interrelationship of the endothelial cells tightly resting on the basement membrane components of the invading capillaries as shown by the spectacular morphological high power images of Trueta's studies (Trueta, 1963).

## Functionalized intrinsically osteoinductive biomimetic matrices spontaneously initiate “*bone: formation by autoinduction*”

A series of studies using coral-derived macroporous constructs in the late eighties did indicate that the spontaneous induction of bone formation is preceded by the induction of mesenchymal tissue condensations (Figures 1A–D). Later bone, often if not always, initiates in concavities of the macroporous spaces of the coral-derived bioreactors (Figures 1M,N). These frequent morphological observations when quantitating the induction of bone formation set into motion the implementation of biomimetic matrices constructed with highly sintered crystalline hydroxyapatite with concavities carved on the planar surfaces (Figure 2A).

As predicted by the morphological observations of bone formation in both coral-derived and sintered highly crystalline hydroxyapatite macroporous constructs, bone initiated exclusively in the concavities as cut in solid highly crystalline sintered hydroxyapatite discs when implanted intramuscularly in *Papio ursinus* [Ripamonti and Kirkbride, 1995 (PCT WO95/3200); Ripamonti et al., 1999; Ripamonti and Kirkbride, 2001; (US6,302,913B); Ripamonti, 2004] (Figures 2B–D).

Pre-clinical studies in *Papio ursinus* have identified that macro-concavities within the macroporous spaces are determinant of bone formation when prepared in calcium phosphate-based biomatrices and implanted in extraskeletal sites [Ripamonti et al., 1993; Ripamonti and Kirkbride, 1995; (PCT WO95/3200); Ripamonti and Kirkbride, 2001 (US6,302,913B 2001)]. Our studies have thus shown that “*the driving force of the intrinsic induction of bone formation by bioactive biomimetic matrices is the shape of the implanted scaffold*” (Ripamonti, 1996, 2004, 2009; Ripamonti et al., 1999, 2007b).

We did previously stated (Ripamonti, 2009) that “*the language of shape is the language of geometry. The language of geometry is the language of a sequence of repetitive concavities that biomimetize the remodeling cycle of the primate cortico-cancellous osteonic bone*” (Parfitt, 1994; Manolagas and Jilka, 1995; Parfitt et al., 1996).

The concavity *per se*, “*as cut into calcium phosphate macroporous constructs or generated by osteoclastogenesis during the remodeling cycle of the primate osteonic bone, is the geometric signal that initiates the induction of bone formation*” (Ripamonti, 2009) as prompted after osteoclastogenesis of either calcium phosphate-based constructs or of the mineralized trabeculae of the cortico-cancellous osteonic bone (Parfitt, 1994; Manolagas and Jilka, 1995; Parfitt et al., 1996).

Concavities are endowed with “*shape memory geometric cues in which soluble molecular signals induce morphogenesis, and physical forces, imparted by the geometric topography of the carrier substratum, dictate biological patterns, constructing the induction of bone formation, and regulating the expression of gene products as a function of the structure*” (Ripamonti, 2006). The connubium of smart biomimetic bioreactors with biologically functionalized surfaces *per se* induces mRNA species of selected gene products. Secreted proteins initiate tissue induction and morphogenesis controlled by functionalized surface' geometries (Figures 2A–D).

A critical experiment that further showed the role of macrophages/osteoclasts in altering the surface characteristics of the implanted macroporous bioreactors followed the harvest and histological analyses of coral-derived constructs with incomplete conversion of the original calcium-carbonate biomatrix (Ripamonti et al., 2009). Bone did not form by day 90 but did form, and substantially so, in heterotopic specimens harvested on day 365. The results after implantation of 5 and 13% partially converted hydroxyapatite/calcium carbonate constructs clearly indicated that “*post-implantation modifications of the substrata were critical for the differentiation of osteoblastic-like cells expressing, secreting and embedding osteogenic molecular signals onto the biomimetic matrices*” (Ripamonti et al., 2009).

Indeed, in recent experiments in *Papio ursinus*, macroporous constructs preloaded with 0.24 mg of the biphosphonate zoledronate Zometa®, that blocks osteoclastic activity, showed very limited induction of bone formation within the macroporous spaces (Ripamonti et al., 2010). These results did show that “*osteoclastic post-implantation modifications of the implanted macroporous substrata are critical for the induction of macro- and micro-patterned topographies highly suitable for the differentiation of osteoblastic-like cells expressing and secreting the osteogenic soluble molecular signals of the TGF-*β *supergene family*” (Ripamonti et al., 2009, 2010).

In further mechanistic experiments, our laboratory did report the critical role of calcium ions (Ca^++^) by implanting macroporous bioreactors in the *rectus abdominis* muscle after preloading the bioreactors with the calcium channel blocker verapamil hydrochloride. Doses of verapamil hydrochloride strongly inhibited bone formation (Klar et al., 2013). Morphological and histomorphometrical analyses showed limited bone formation together with disorganized tissue patterning without the induction of collagenous condensations resulting in lack of bone formation (Klar et al., 2013). Molecularly, there was down-regulation of *BMP-2* but up-regulation of *Noggin*, responsible to the very limited, if any, formation of bone (Klar et al., 2013; Ripamonti et al., 2015).

The concavity is thus conducive to induce a confined and protected micro-environment within the macroporous substratum that enables resident perivascular/myoblastic stem cells to differentiate into osteoblastic-like cells after nanotopographic geometric modifications. Ca^++^ release sets into motion angiogenesis with capillary sprouting, and the induction of osteogenic mRNA species of the TGF-β supergene family, later embedded as gene products onto the hydroxyapatite surface of the inductive concavity (Ripamonti et al., 1999, 2014, 2015; Klar et al., 2013; Figures 2F,G).

**Figure 3:**
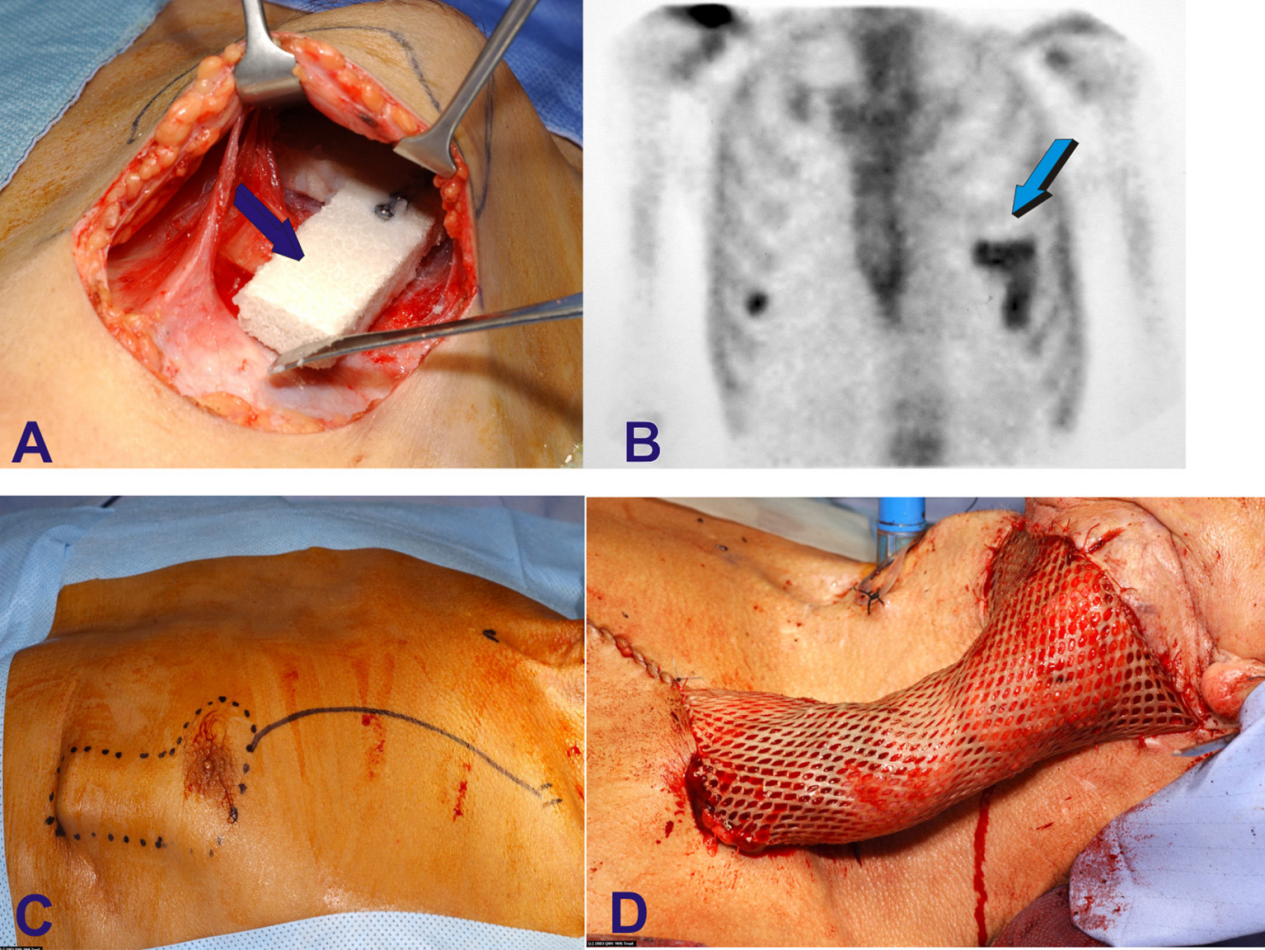
Human intramuscular heterotopic fabrication of a hydroxyapatite/bone morphogenetic protein-7 (hBMP-7) bioreactor for auto-transplantation to large mandibular defect after squamous cell carcinoma avulsion and debridement. The compelling problem of limited osteoinduction in spite of supra-physiological doses of human recombinant morphogenetic protein-2 and -7 (hBMP-2, hBMP-7) forced the use of heterotopic vascularized muscular sites to construct pre-fabricated osteogenic implants for later auto-transplantation in orthotropic mandibular site in a human patient (Heliotis et al., 2006). **(A)** After the analyses of several coral-derived macroporous constructs implanted in the *rectus abdominis* muscle of *Papio ursinus* (Ripamonti, 1991, 1996, 2006; Ripamonti et al., 1993, 2009, 2010) a L-shaped coral-derived construct was also implanted into the *pectoralis maior* of a human patient combined with 2.5 mg doses of hBMP-7 (Heliotis et al., 2006). **(B)** Scintigraphy shows bone formation within the prefabricated intramuscular construct later **(C)** rotated with a composite myo-cutaneous flap **(D)** into the recipient autologous mandibular bone defect (Heliotis et al., 2006).

The concomitant compelling problem of recombinant human bone morphogenetic proteins application in human patients with lack of translational efficacy into clinical contexts (Ripamonti et al., 2006, 2007a, 2012b; Williams, 2006; Department of Justice, 2009; Carragee et al., 2011a; Centre for Devices and Radiological Health, 2011; Fauber, 2011a,b) did invocate the use of a healthy vascularized heterotopic muscular site to induce a prefabricated coral-derived/osteogenic protein-1 (hOP-1) bioreactor transported with a vascularized pedicled bone flap in the human chest to repair a large mandibular defect (Figure 3; Heliotis et al., 2006). The study showed that hOP-1, combined with a large coral-derived construct mimicking the human mandible in the chest of the patient did result in heterotopic induction of bone formation in man, and “*without the addition of cortical bone, bone marrow aspirates or any other bone precursor*” (Heliotis et al., 2006).

## Osteoinductive hydroxyapatite-coated titanium implants spontaneously induce the induction of bone formation

**Figure 4:**
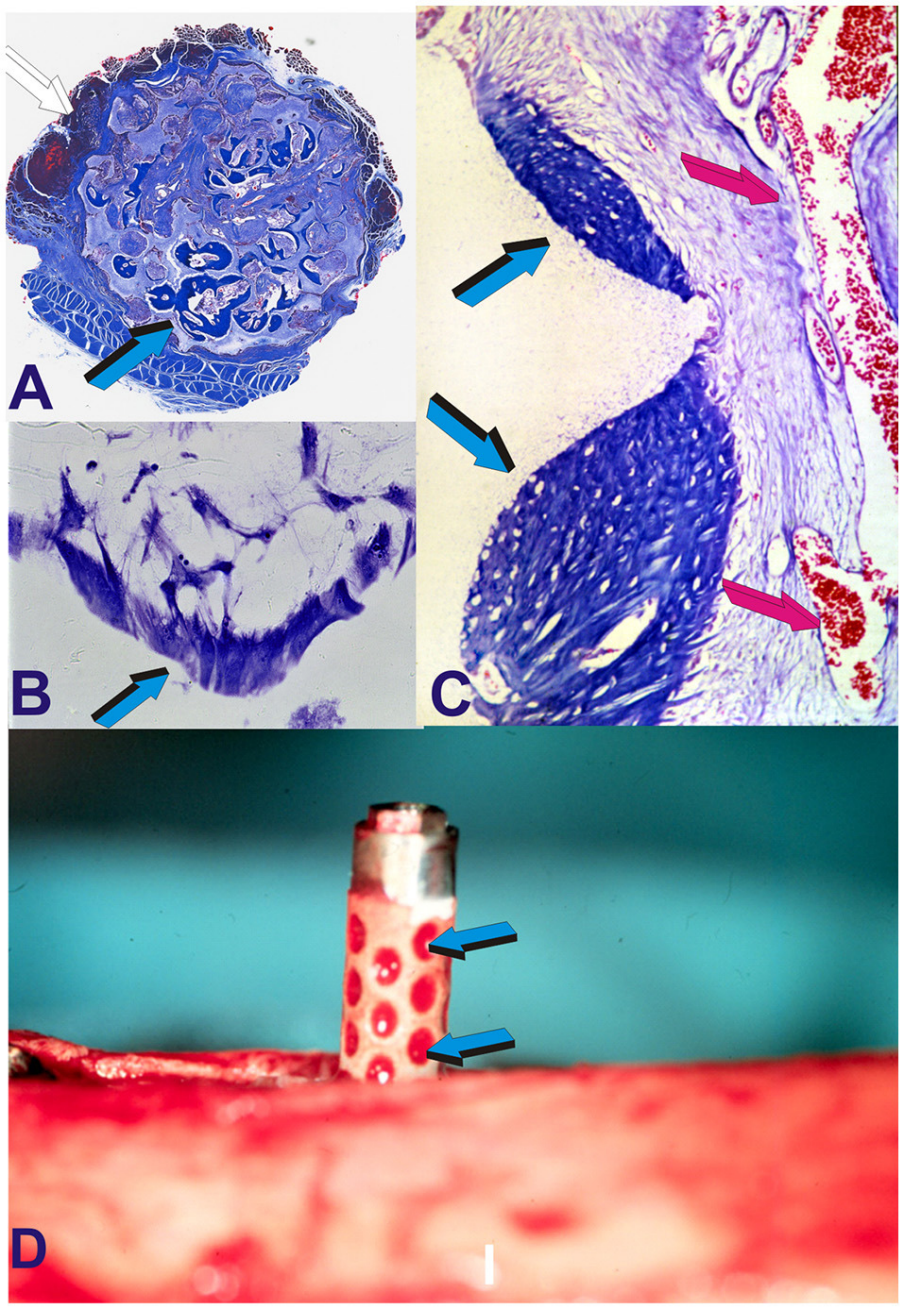
Molecular, morphological and biological conceptualization of the role of the concavity to construct intrinsically osteoinductive hydroxyapatite-coated titanium implants for the spontaneous induction of bone formation. **(A)** Substantial induction of bone formation (light blue arrow) by highly sintered highly crystalline hydroxyapatite bioreactors implanted in the *rectus abdominis* muscle of the Chacma baboon *Papio ursinus* (Ripamonti et al., 1999). **(B)** Alignment and orientation of MC 3T3-E1 pre-osteoblastic cells within a concavity (light blue arrow) of the coral-derived bioreactor *in vitro* (Ripamonti et al., 2012a). **(C)** Digital image of a macroporous sintered hydroxyapatite resembling the tread of an implant implanted heterotopically yet spontaneously inducing bone (light blue arrows) within the concavities of the bioreactor in close relationship with invading capillaries (magenta arrows). **(D)** The “*concavity motif*,” the “*concavity: the shape of life*” (Ripamonti, 2006; Ripamonti et al., 2012a) is then re-assembled in a titanium construct prepared with concavities along the substratum later coated by highly crystalline plasma sprayed sintered hydroxyapatite onto the prepared titanium surface (Ripamonti et al., 2012a). Light blue arrows **(D)** indicate the adsorption within concavities of plasma products during the surgical implantation in orthotopic mandibular and tibial sites (Ripamonti et al., 2012a).

The clinical and pre-clinical translation of the “*geometric induction of bone formation*” (Ripamonti et al., 1999; Ripamonti, 2004) was set after evaluating the induction of bone formation across both sintered macroporous constructs and solid discs of highly sintered crystalline hydroxyapatite on day 30 and 90 after heterotopic intramuscular implantation in the *rectus abdominis* of *Papio ursinus* (Ripamonti et al., 1999; Ripamonti, 2004; Figures 1, 2). The digital images shown in Figures 1L–N and Figures 4A–C set into motion the design, manufacturing and testing of geometrically-constructed hydroxyapatite-coated titanium implants. The ultimate goal was to produce a geometrically intrinsically *per se* osteoinductive hydroxyapatite plasma-sprayed-titanium implant for therapeutic clinical translation [Figure 4D; Ripamonti and Kirkbride, 2001; Ripamonti et al., 2012a; (US6,302,913 B1)].

Of great interest, studies reported the induction of bone formation by macroporous titanium implants 12 months after heterotopic implantation in the dorsal musculature of canines (Fujibayashi et al., 2004). Significantly, the authors reported that the “*in vitro apatite-forming ability*” may be a necessary pre-requisite for macroporous titanium implants to be osteoinductive (Fujibayashi et al., 2004). Bioactive titania might have formed a thin coating of calcium phosphate material *in vivo*. As BMPs do have high adsorption capacity for hydroxyapatite (Ripamonti et al., 1993; Reddi, 2000; Ripamonti, 2006), titanium apatite-forming ability might have adsorbed locally produced BMPs later initiating the induction of bone formation as a secondary response (Ripamonti et al., 2012a). In further experiments, Fujibayashi' group studied osteoinduction in macroporous titanium blocks following sodium removal by dilute HCl treatment (Takemoto et al., 2006), as well as titanium blocks after titanium surfaces prepared by alkali and heat treatment (Fujibayashi et al., 2001). Of note, however, titanium constructs as used above were macroporous blocks with *in vitro* apatite-forming ability (Fujibayashi et al., 2004).

As discussed in previous communications (Ripamonti et al., 2012a), can bone be formed spontaneously by uncoated titania' substrata when heterotopically implanted in different animal models? The work of Fujibayashi' group only reported data on titania' macroporous constructs with superior *in vitro* as well as *in vivo* apatite-forming ability (Fujibayashi et al., 2004) which may have bound locally-secreted BMPs to the site of surgical implantation. Regardless, studies on BMPs binding capacity to titania' substrata are not available, and in spite of the reported work on macroporous titania' constructs (Fujibayashi et al., 2004; Takemoto et al., 2006), the spontaneous and/or intrinsic osteoinductivity of solid titanium constructs with or without geometric configurations is yet unproven.

Concavities biomimetize the remodeling cycle of the cortico-cancellous osteonic primate bone (Parfitt, 1983, 1994; Manolagas and Jilka, 1995; Parfitt et al., 1996) thus initiating the bone formation phase by recruiting perivascular/pericytic stem to be differentiated into osteoblastic-like cells secreting bone matrix at the hydroxyapatite interface. The remodeling cycle of the cortico-cancellous bone requires “*resting*” periods, with quiescent cells over the trabeculae, “*activation*” periods, whereby activated osteoclasts resorb the mineralized bone matrix, and “*formation*” phases, whereby differentiated osteoblasts develop in the concavity lacunae cut by activated osteoclasts to finally secrete and deposit new bone matrix within the “*concavities*” as cut by osteoclastogenesis (Parfitt, 1994; Manolagas and Jilka, 1995; Parfitt et al., 1996).

**Figure 5:**
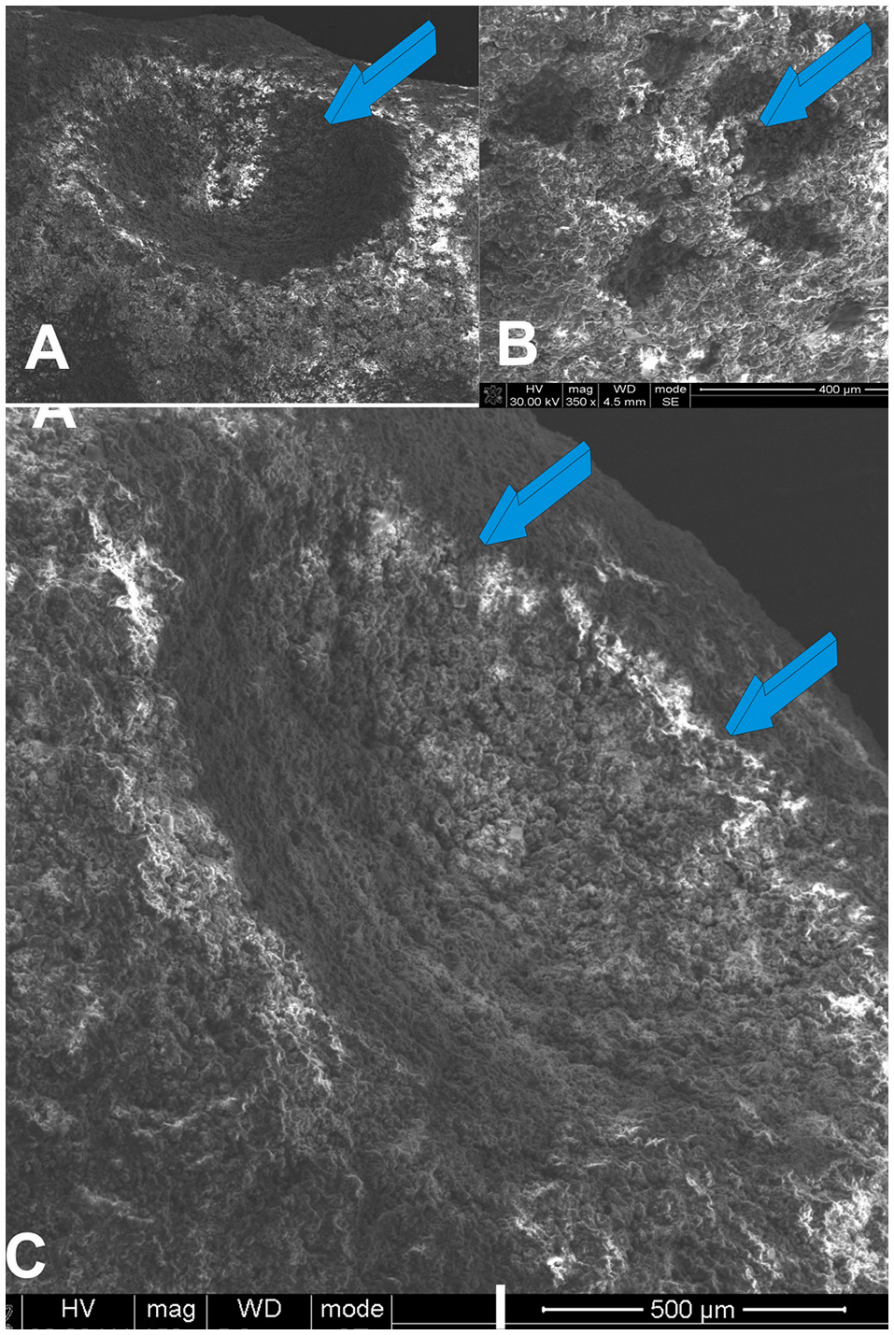
Scanning electron microphotographs (SEM) of the geometrical configuration of the concavity assembled into the titanium bioreactors. **(A,B)** Macro- and microporous geometric landscapes of titania substrata later coated by highly sintered crystalline hydroxyapatite. Macro- and micro-topography of the constructed concavities (light blue arrows) within the titanium' bioreactors present surface topographies that result I the induction of bone differentiation *in vivo* (Ripamonti et al., 2012a, 2013). **(C)** A concavity (light blue arrows) prepared on the titanium' surface show nano-topographic macro- micro-porosities that are inductive of cell differentiation and the expression of the osteogenic phenotype (Ripamonti et al., 2012a, 2013).

The geometric induction of bone formation has been then translated to fabricate geometrically inducive titanium dental implants coated by plasma spraying highly crystalline sintered hydroxyapatite (Figures 4, 5). The coating was applied by air-spraying of high crystallinity, low porosity, highly adherent hydroxyapatite, 30 μm in thickness (Ripamonti et al., 2012a). Prior to spraying with the sintered hydroxyapatite (powder supplied by Metco-Plasma Technick, product AMDRY 650), titania were roughened by grit blasting with alumina grit [Ripamonti and Kirkbride, 2001 (US 6,302,913B1)]. Detailed methodologies and specific fabrication techniques are listed in published US patents [Ripamonti and Kirkbride, 2001 (US 6,302,913B1)]. As previously reported, the concavities as prepared on the titanium implants, generate *in vivo* bioreactors along the titanium profile as a space between the drilled rotary instrumented bone surfaces as well as the enveloping *rectus abdominis* muscle. Titania constructs were implanted orthotopically in edentulous mandibular ridges and anterior medial region of the surgically exposed tibiae, and in the *rectus abdominis* muscle of adult baboons, respectively [Ripamonti and Kirkbride, 2001 (US 6,302,913B1); Ripamonti et al., 2012a].

Adult *Papio ursinus* animals were euthanized on day 30 and 90 and undecalcified specimen blocks harvested and processed in ascending concentrations of Technovit 7200 VLC (Heraeus Kulzer GmbH, Wehrheim, Germany). All samples' preparation was performed using the EXAKT precision cutting diamond saw and grinding system (EXAKT Apparatebau, Nordestedt, Hamburg, Germany) (Ripamonti et al., 2012a, 2013).

**Figure 6:**
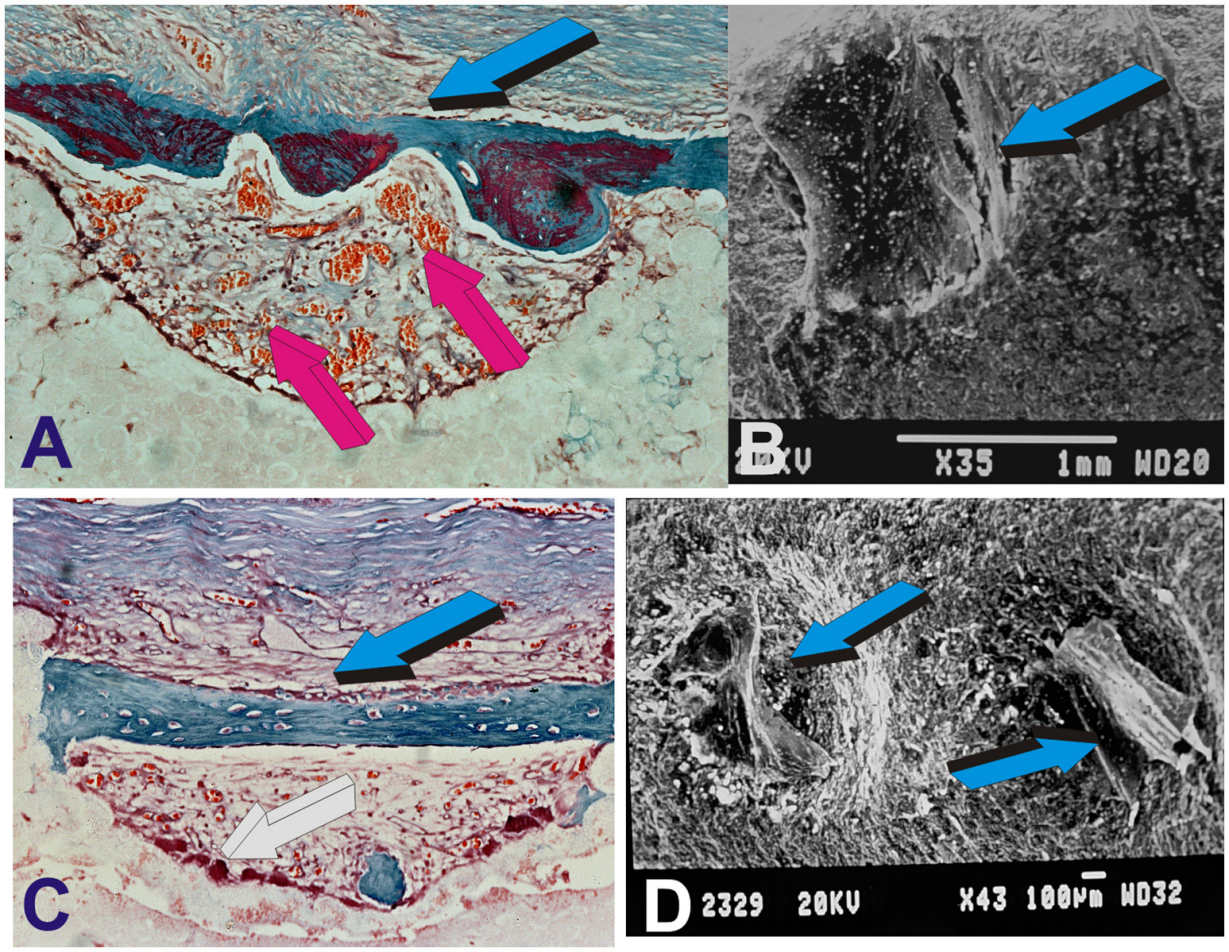
The concavity, the shape of life, and the driving morphogenetic force of the geometric induction of bone formation. **(A,C)** Concavities, prepared in solid monolithic discs of sintered highly crystalline hydroxyapatite (Ripamonti et al., 1999, *de novo* generate intramembranous bone bridges (light blue arrows in **A**) with pronounced vascular invasion and angiogenesis (magenta arrows). **(B,D)** 5 days after heterotopic implantation of titanium constructs as shown in Figure 4D, fibroblast-like cells synthesize collagen fibers across the edges of the concavities implanted in the *rectus abdominis* muscle of *Papio ursinus*. Fibroblast-like cells and/or myoendothelial cells synthesize collagen fibers migrating whilst synthesizing back and forward from the edges of the concavities (light blue arrows in **B**) thus assembling extracellular matrix **(C)** for later mineralization and transformation into bone as shown in **(A,C)**.

One animal needed to be euthanized 5 days after heterotopic implantation. Heterotopic constructs were harvested from the *rectus abdominis* muscle for scanning electron microscopy (SEM) preparation and analyses [Ripamonti and Kirkbride, 2001 (US 6,302,913B1); Ripamonti et al., 2012a, 2013]. SEM analyses on day 5 showed multicellular driven cellular patterning and organization along the geometric implants as compared to standard linear constructs. Concavities resulted in selected spatial organization and patterning of the invading myoblastic/pericytic stem cells. There was induction of cellular trafficking, palisading with crossing collagenous bundles across the edges of the concavities (Figures 6B,D) as early as 5 days after heterotopic implantation, later self-organizing in mineralized bone bridges surfaced by osteoid with secreting osteoblasts across the heterotopic geometric bioreactors (Figures 6A,C).

**Figure 7:**
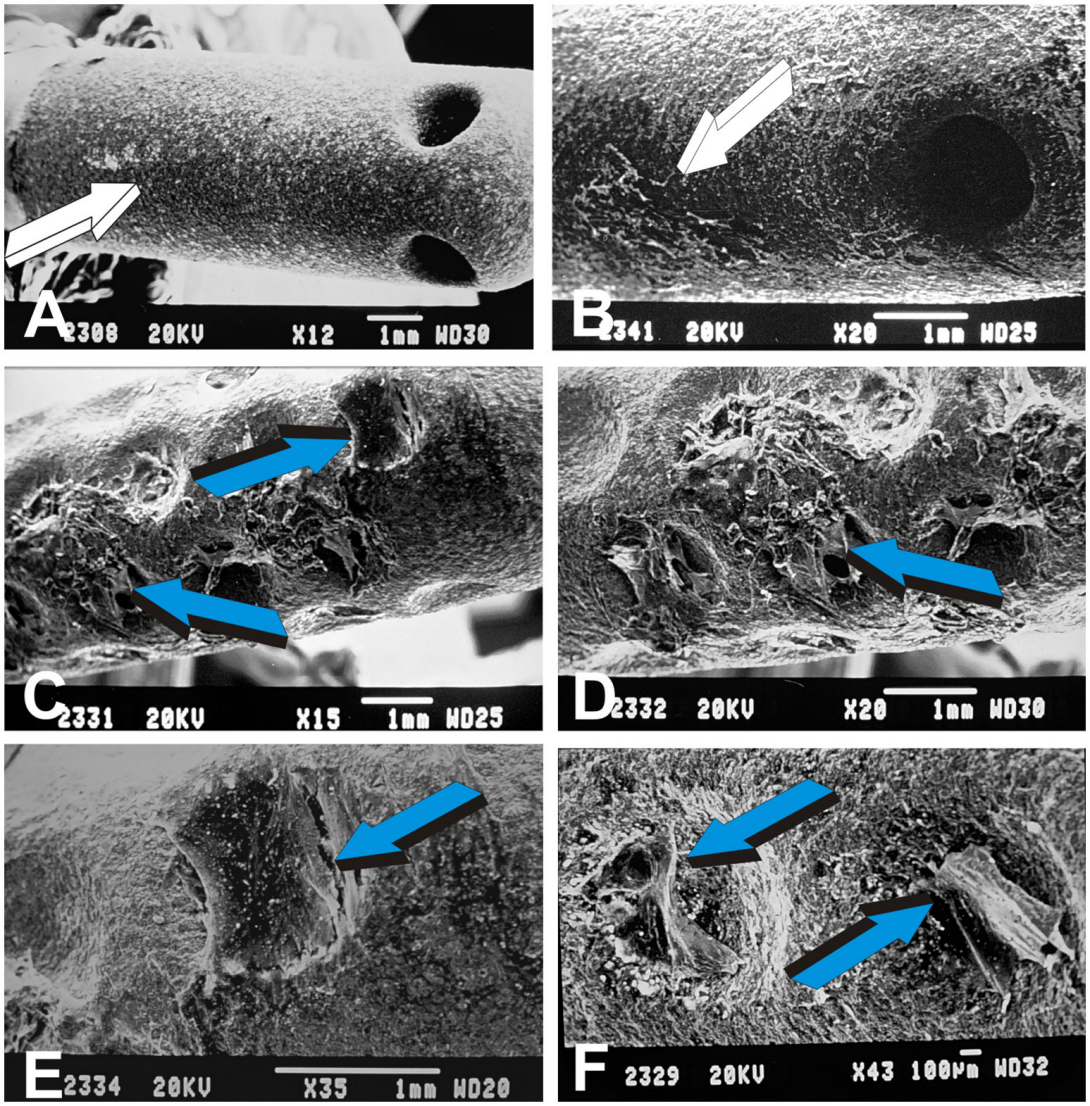
Morphogenetic landscape of the geometric induction of tissue morphogenesis and tissue patterning by specific geometric configurations of titanium substrata coated with highly crystalline high adhesion sintered hydroxyapatite harvested 5 days after *rectus abdominis* implantation. **(A,B)** Lack of cellular differentiation and trafficking (white arrows) on planar control hydroxyapatite-coated titanium substrata. **(C,D)** Hyper cellularity, cellular patterning and cellular assembling (light blue arrows) along concavities of the titanium bioreactor, underlying the morphogenetic and inductive capacity of “*the concavity: the shape of life*“ (Ripamonti, 2006). **(E,F)** Tissue patterning and assemblages of tractional collagenic gradients across the concavities by synthesized collagen fibers from edge to edge of the elevated margins of the concavities, later transformed into intramembranous bone bridges across concavities of the highly sintered crystalline constructs as shown in **(A,C)**.

Figure 7 summarizes the biological inductive landscape of the concavity' bioreactor showing multiple cellular activities, trafficking and palisading across the edges of the concavities of the geometric implants (Figures 7C–F). In marked contrast, there is limited activity, if any, on standard planar hydroxyapatite coated implants (Figures 7A,B).

**Figure 8:**
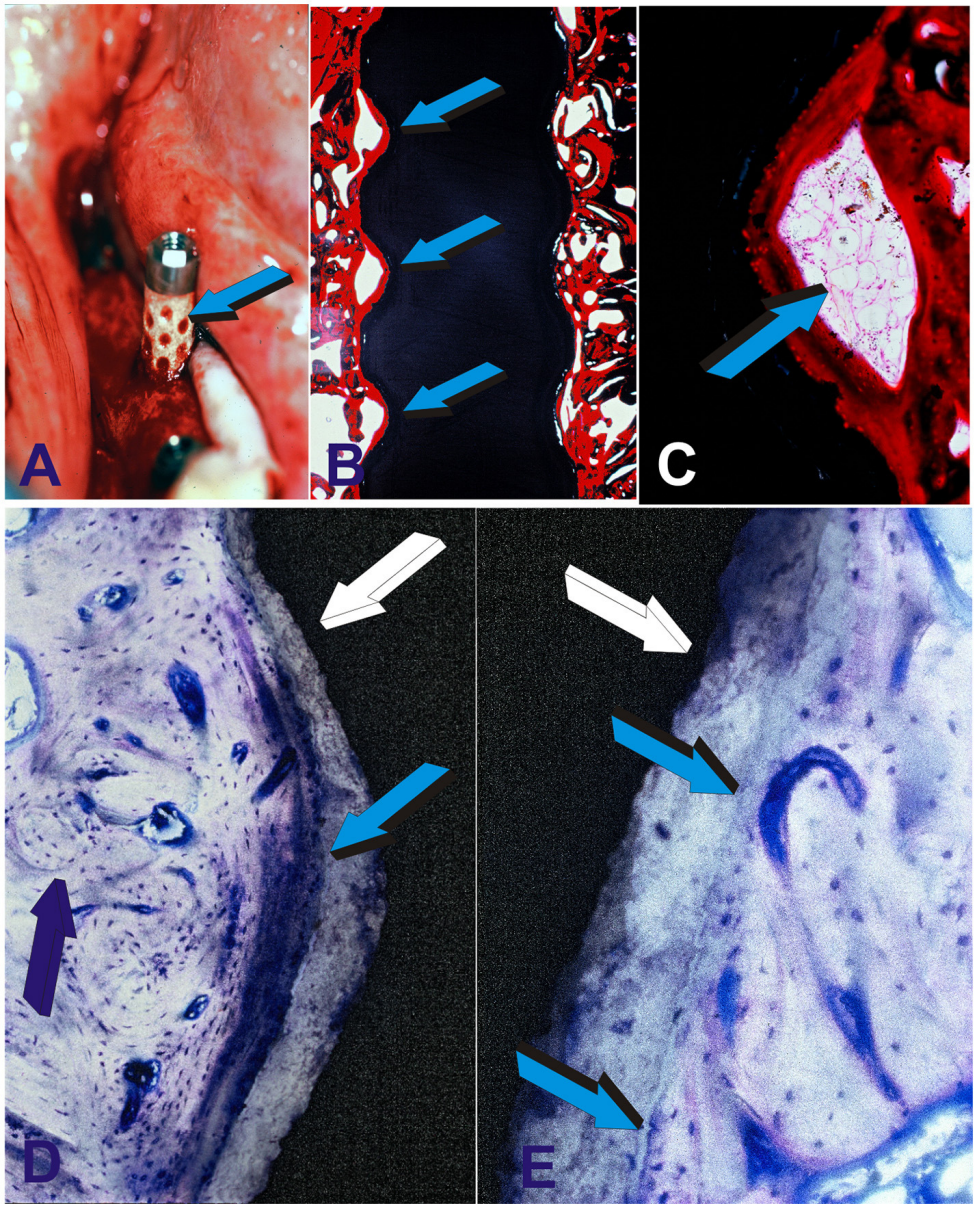
Preclinical testing of the geometric self-inductive constructs in edentulous mandibular ridges of adult Chacma baboons *Papio ursinus*. **(A)** Highly crystalline hydroxyapatite coated titanium geometric constructs are implanted in edentulous ridges of adult *Papio ursinus*. Note the selected concentration/adsorption of plasma and plasma products onto the concavities of the geometric implant (light blue arrow). Binding of plasma and/or serum material onto the concavities may possibly act as a reservoir of plasma factors including fibrin and fibronectin for **(B)** early attachment along the severed bone inducing continuous osteointegration with newly formed bone tightly attached along the concavities of the implanted bioreactor with **(C)** remodeling bone with marrow within the inductive concavity of the implanted bioreactor. **(D)** Tight osteointegration to the sintered hydroxyapatite coated titanium construct (light blue arrow) with newly formed lamellar osteonic bone (dark blue arrow) tightly attached to the plasma sprayed hydroxyapatite coating onto the titanium substratum (white arrow). **(E)** High power view digital image showing the tight and remodeled fusion and osteointegration of the newly formed synthesized bone within a concavity of the titanium bioreactor (white arrow) coated by plasma sprayed highly crystalline sintered hydroxyapatite. Light blue arrows indicate the tight integration and biological osteointegrated blending of the newly formed bone within the concavity onto the plasma sprayed hydroxyapatite facing the lamellar osteonic bone with multiple osteocytes and capillaries along the remodeled newly formed bone matrix.

On day 30 and 90 after orthotopic mandibular and tibiae implantation, geometrically-driven titania constructs showed superior osteointegration [Figure 8; Ripamonti and Kirkbride, 2001 (US 6,302,913B1); Ripamonti et al., 2012a, 2013]. Remodeled bone was tightly integrated to the plasma-sprayed hydroxyapatite coating, merging with the remodeled osteonic bone that initiated within the concavities' bioreactors (Figures 8D,E).

**Figure 9:**
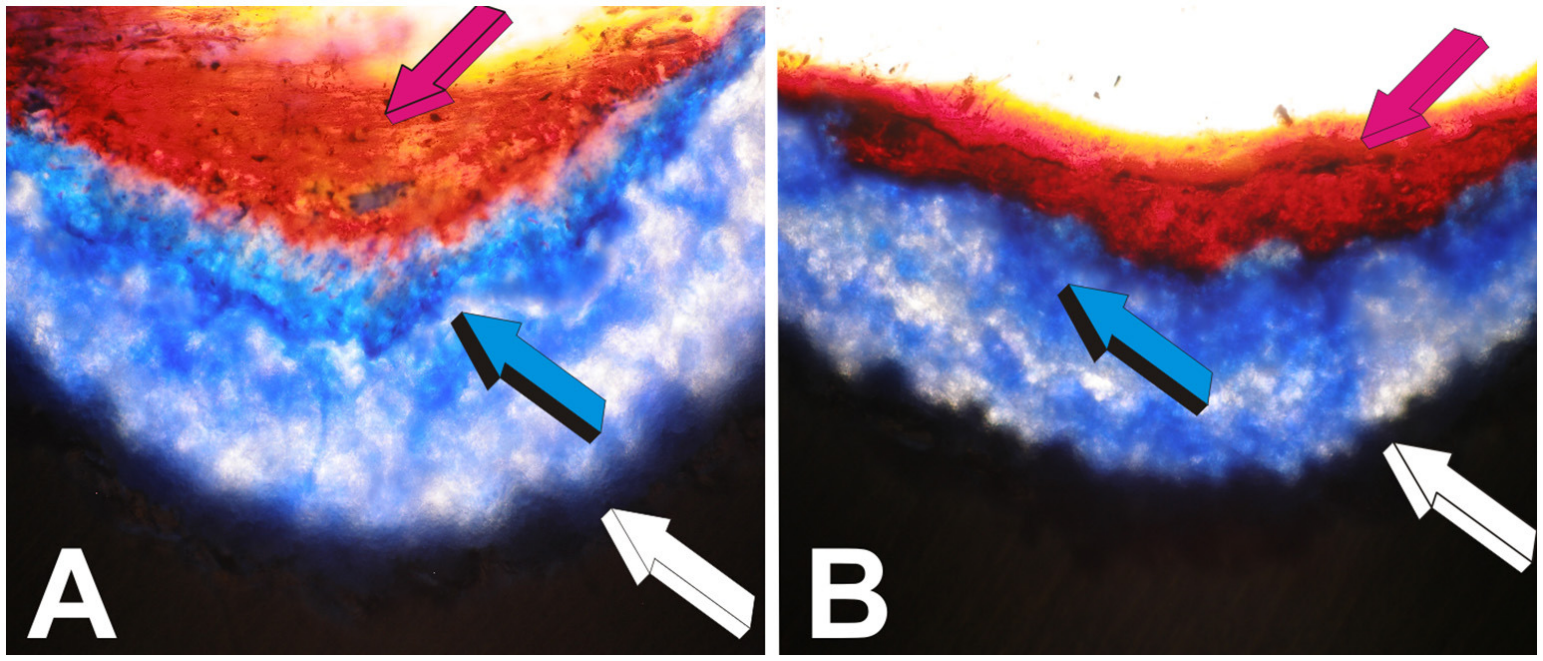
The crux of the problem of bone tissue engineering and regenerative medicine in clinical context. Can we engineer an activated bioreactor that spontaneously, and/or intrinsically initiate the ripple-like cascade of bone differentiation by induction, without the exogenous application of the osteogenic soluble molecular signals of the transforming growth factor-β (TGF-β) supergene family? (Ripamonti, 2003) and when implanted in human patient synthesize *per se* “*Bone: formation by autoinduction*?” (Urist, 1965). The undecalcified images presented in **(A,B)** show that the above is possible, that hydroxyapatite coated titanium geometric constructs *per se* initiate the spontaneous induction of bone formation in primate models (Ripamonti et al., 2012a) and without the exogenous application of the osteogenic soluble molecular signals of the TGF-β supergene family. **(A)** High power view of a concavity of hydroxyapatite-coated titanium construct (white arrow) harvested from the *rectus abdominis* muscle of *Papio ursinus*. Light blue arrow indicates the induction of mineralized newly formed bone (darker blue) surfaced by an osteoid seam (magenta arrow) surfacing the mineralized bone. **(B)** In another inductive concavity identical morphological patterns of induction with newly formed mineralized bone (light blue arrow) surfaced by a large osteoid seam. Undecalcified sections cut at 30 μm using the Exakt diamond saw grinding and polishing equipment (Ripamonti et al., 2012a).

Harvested constructs from the *rectus abdominis* muscle 31 months after implantation (2.7 years) showed the induction of bone formation within concavities of the heterotopically implanted bioreactors (Figure 9; Ripamonti et al., 2012a, 2013). Experimental research from the bench top to pre-clinical experimentation in primate models has thus resulted in the development and construction of the only reported hydroxyapatite-coated titanium dental implant that *per se* is intrinsically osteoinductive when implanted in intramuscular sites where there is no bone [Ripamonti and Kirkbride, 2001 (US 6,302,913B1); Ripamonti et al., 2012a, 2013]. Biomimetic nano-topographic geometries carved on titanium implant for craniofacial and/or orthopedic rehabilitation “*re-program somatic stem cells to initiate osteogenic differentiation and the induction of bone* formation” [Ripamonti and Kirkbride, 2001 (US 6,302,913B1); Ripamonti et al., 2012a, 2013].

How do nanopatterned surface topographies and geometry invocate the induction of the osteogenic phenotype? How structure/function relationships are mechanistically driven, and how to resolve and assign the structure/activity profile?

The role of implant' surface geometry on the induction of the osteogenic phenotype was studied by Chehrouidi et al. in the early nineties (Chehroudi et al., 1992). The study showed that implant' geometry and topography directly control the induction of the osteogenic phenotype both *in vitro* and *in vivo* (Chehroudi et al., 1992). Kuboki' group reported that the geometry of carrier substrata controls the phenotypic expression of BMPs-induced osteogenesis (Kuboki et al., 2001). The study goes further stating that the reported data “*propose a classification of geometry of the artificial extracellular matrices that is useful for designing a scaffold for tissue engineering of bone and related tissues*“(Kuboki et al., 2001).

Which are the molecular mechanisms whereby patterned surface geometries and topographies invocate the induction of the osteogenic phenotype setting into motion the induction of bone formation? We have previously stated that “*the innovation of re-programming somatic stem cells to induce osteogenic differentiation and the induction of bone formation*” (Ripamonti et al., 2012a) has provided mechanistic strategies to induce and maintain osteointegration around titanium implants for craniofacial and orthopedic applications. The key word is thus induction, that is a biomimetic matrix that *per se* transforms resident responding stem cells into osteoblastic-like cells *de novo* secreting the induction of bone formation, and without the addition of the osteogenic soluble molecular signals of the TGF-β supergene family (Ripamonti et al., 1993, 1999, 2012a, 2014; Klar et al., 2013).

Functionalization of distinct titania nanotopographies with controlled nano features initiating osteogenesis has been reported by culturing mesenchymal stem cells on titanium substrata patterned with nanopillar structures of 15, 55 or 90 nm high nanopillars (McNamara et al., 2010, 2011). It was demonstrated that “*the cell response to the 15 nm high nanopillars was distinct from the other nanofeatured surfaces, with cell displaying larger focal adhesions, increased levels of the osteogenic transcription factor phospho-Runx2 and greater expression of osteocalcin“* (McNamara et al., 2010, 2011).

Though several groups confirmed the spontaneous and/or intrinsic osteoinductivity (Ripamonti, 1996) of a variety of calcium phosphate-based biomaterials (Vargervik, 1992; Yamasaki and Sakai, 1992; Toth et al., 1993; Yuan et al., 2000, 2001, 2006, 2010; Gosain et al., 2002, 2004; Habibovic et al., 2005, 2006a,b, 2007, 2008; Le Nihouannen et al., 2005; Habibovic and de Groot, 2007; Li et al., 2008; Barradas et al., 2011; Davinson et al., 2014, 2015; Danoux et al., 2016), mechanistic insights were however lacking, and a plethora of hypotheses were invocated to resolve the spontaneous induction of intramembranous bone formation (references above, and Ripamonti et al., 2009 for review). Hypotheses included the critical role of hydroxyapatite' nano-topography, crystallinity, 3D sintering temperatures, structural properties, and optimal micro porosity (references above, and Ripamonti et al., 2009 for review).

The immunohistochemical evidence of BMPs-gene products embedded at the hydroxyapatite interface of the concavity (Ripamonti et al., 1999) did indicate for the first time a mechanistic insight into the spontaneous induction of bone formation by macroporous hydroxyapatites (Ripamonti et al., 1999). This research work however could not differentiate between osteogenic proteins either locally produced or circulating and later adsorbed, or embedded, onto the permissive hydroxyapatite concavity' microenvironment, thus initiating bone formation as a secondary response (Ripamonti et al., 1999).

Later studies showed that mRNA of osteogenic protein-1 (OP-1) is expressed within the concavities by differentiating resident stem cells (Ripamonti et al., 2007b). Secreted gene products are later embedded within the regulatory *smart* concavities of the substratum (Ripamonti et al., 2007b). Molecular work did show that the initiators of the spontaneous and/or intrinsic induction of bone formation are the expressed and secreted BMPs gene products, reporting *BMPs* genes and particularly *BMP-2* expression by the regulatory macroporous constructs (Klar et al., 2013). More elegantly perhaps, preloading coral-derived constructs with doses of recombinant human Noggin (hNoggin), a BMPs inhibitor, showed minimal, if any, induction of bone formation, indirectly indicating that the secreted BMPs gene products had been blocked by recombinant hNoggin, resulting in lack of bone differentiation (Klar et al., 2013; Ripamonti et al., 2015).

When browsing the surgical experimental record book (Ripamonti, 1989; NIH Federal Supply Service 7530-222-3525) on coral-derived macroporous calcium phosphate-based constructs, we read, as written in 1989, that “*the formation of tight mesenchymal aggregates or condensations is the earliest morphogenetic event associated with the position-dependent differentiation of skeletal structures in the developing vertebrate limb*.” We then further stated that “*the condensation or assemblage of collagenous matrix is a substrate that presages morphological differentiation of an intramembranous pattern of bone development*.” We did further postulate “*the assumption that the condensations are organized by matrix proteins*” and that “*presumably osteogenin and related bone morphogenetic proteins trigger the morphological differentiation of the osteogenic phenotype*.”

Several years have elapsed when working on an NIH Record Book and at last our recent studies did show limited tissue patterning with lack of bone differentiation in coral-derived macroporous bioreactors when recombined with 150 μg recombinant hNoggin (Ripamonti et al., 2015). Morphological examination did show limited tissue patterning with poorly remodeled connective tissue matrix, and limited vascular invasion. Of note, there were haphazardly patterned tissue condensations with limited capillary invasion, resulting in lack of bone formation (Ripamonti et al., 2015). Our hypothesis then presented in the 1989 NIH record book was thus confirmed by the morphological and molecular findings that hNoggin and Noggin expression would not only inhibit the induction of bone formation (Klar et al., 2013; Ripamonti et al., 2015) but more importantly would jeopardize the induction of collagenous condensations thus implying a direct role for BMPs in setting the induction of collagenous condensations as a prerequisite for the later induction of bone formation. This has been an important finding of our molecular studies (Klar et al., 2013; Ripamonti et al., 2015), implying a critical role for *BMPs* gene and gene products in tissue patterning long before the induction of bone formation.

Molecularly, the addition of hNoggin to the coral-derived bioreactors down-regulates *BMP-4, BMP-6, BMP-7* with however limited up-regulation of *BMP-2* and substantial up-regulation of *BMP-3* (Ripamonti et al., 2015), once again highlighting the pleiotropic gene expression and activation up- and down-regulated by treated and untreated coral-derived bioreactors when implanted in the *rectus abdominis* muscle of *Papio ursinus* to temporally and spatially regulate the induction of bone formation (Ripamonti et al., 2014, 2015).

**Figure 10:**
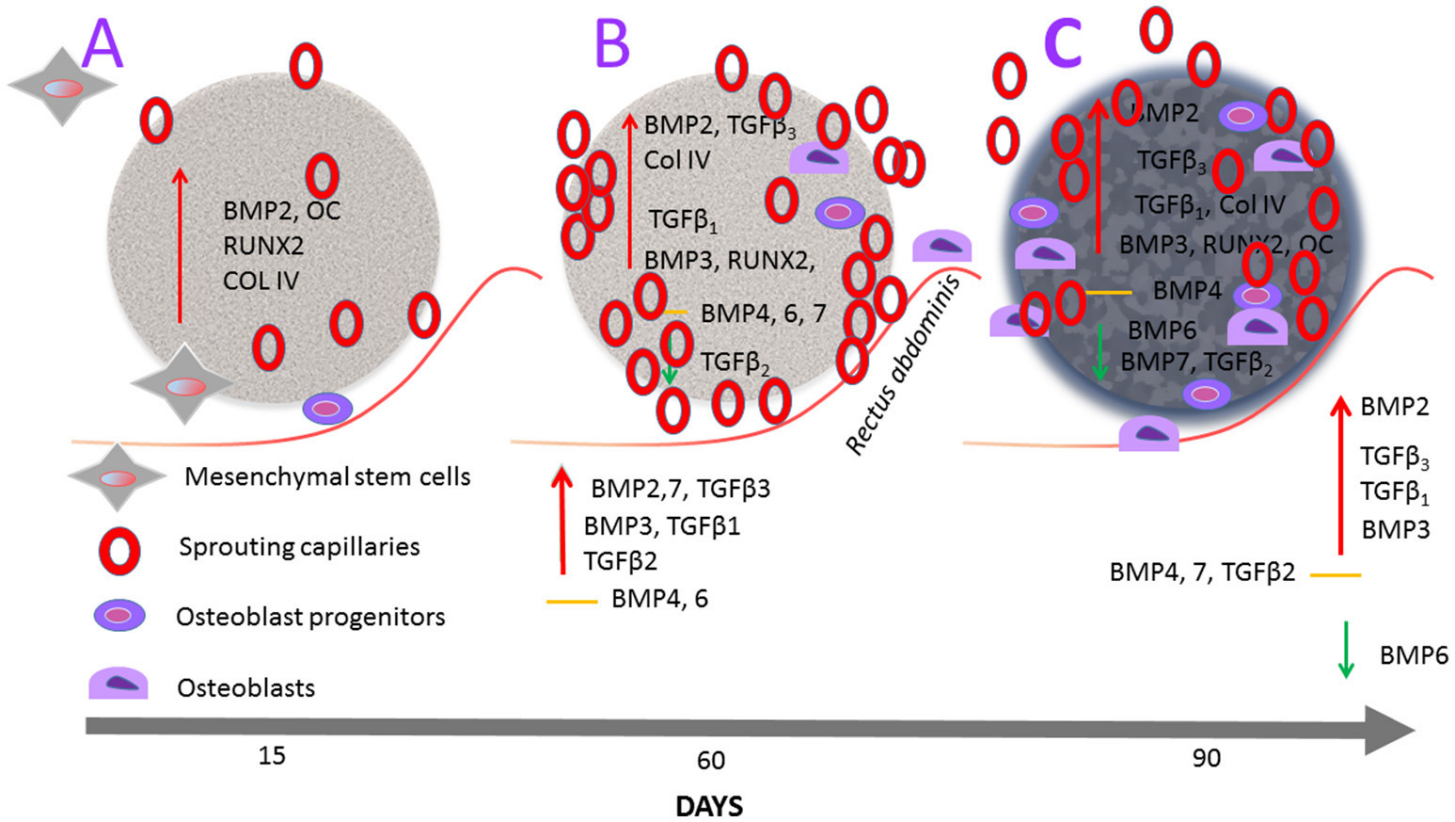
Schematic representation of the hydroxyapatite/induced osteogenesis model by coral-derived macroporous bioreactors *solo* when implanted in the striated *rectus abdominis* muscle of *Papio ursinus*. Coral-derived macroporous constructs are implanted in the *rectus abdominis* muscle (undulated red line) of the Chacma baboon *Papio ursinus*. Constructs are harvested on days 15, 60, and 90 after heterotypic implantation. Undecalcified specimen blocks are embedded in Technovit and sections cut, ground, and polished at 37 μm by the Exakt diamond saw cutting and polishing equipment. **(A)** There is *Runx-2, Osteocalcin, BMP-2* and *Collagen type IV* expression within the coral derived construct with morphologically evident sprouting capillary at the periphery of the implanted construct as well as within the macroporous spaces (Klar et al., 2014). There is however minimal if any expression of selected genes within the surrounding enveloping striated muscle, once again indicating that the primary events of the hydroxyapatite induced osteogenic model predominately are set within the macroporous spaces. **(B)** On day 60, there is further angiogenesis sustained by continuous *Type IV collagen* over-expression together with *TGF-*β_*1*_ and TGF-β_*3*_ over-expression with however *TGF-*β_*2*_ downregulation. Interestingly, there is up-regulation of *BMP-3* as well as expression of *BMP-4, BMP-6* and *BMP-7*. Of note, the surrounding muscle tissue on day 60 expresses *BMP-2, BMP-3*, and *BMP-7* with *TGF-*β_*1*_,*TGF-*β_*2*_, and *TGF-*β_*3*_ with no expression of *BMP-4* and *BMP-6*. **(C)** On day 90, the homogenized coral-derived construct without the enveloping striated *rectus abdominis* muscle show continuous up-regulation of *RUNX-2, Osteocalcin* and *Collagen type IV*, together with up-regulation of *TGF-*β_*1*_ and *TGF-*β_*3*_ with downregulation of *TGF-*β_*2*_*, BMP-6* and *BMP-7*. The enveloping *rectus abdominis* muscle showed up-regulation of *TGF-*β_*1*_, *TGF-*β_*3*_, BMP-2, and BMP3 with down regulation of BMP-6. Molecular pathways correlated with the florid induction of bone formation within the macroporous spaces as shown in Figure 1.

Figure 10 schematically represents the molecular and morphological *connubium* of the reconstructed molecular and morphological events upon the implantation of coral-derived macroporous bioreactors in heterotopic sites of the *rectus abdominis* striated muscle of *Papio ursinus*. The primary differentiating events initiating the induction of bone formation within untreated coral-derived constructs develop first within the macroporous spaces after capillary sprouting and invasion. There is minimal or lack *BMP-2* expression within the surrounding *rectus abdominis* muscle, with however *RUNX-2, Osteocalcin, BMP-2* and *Type IV collagen* expression within the macroporous bioreactors from day 15 to 30, at which time *TGF-*β_*1*_, *TGF-*β_*3*_ are overexpressed with interestingly however *TGF-*β_*2*_ downregulation, suggesting an as yet unknown mechanism of for the induction of bone formation in primates. By day 90, there is further vascular invasion and capillary sprouting hallmarked by *Type IV collagen* expression together with *BMPs* overexpression, particularly *BMP-3* with however downregulation of *BMP-6* and *BMP-7*, highlighting the *TGF-*β and *BMP* genes finely tuning the pathways of the hydroxyapatite-induced osteogenesis model in *Papio ursinus*.

We previously stated that the initiation of bone formation by coral-derived bioreactors solo must “*thus proceed via surface modifications of the highly crystalline calcium carbonate/hydroxyapatite construct which ultimately is the self-constructor of the induction of bone formation*” (Ripamonti et al., 2015). An important step is the vascular invasion and capillary sprouting not only surrounding the coral-derived construct (Ripamonti et al., 1993) but also within the macroporous spaces as prompted by osteoclasts-driven nanotopographical modifications with Ca^++^ release and cellular differentiation, and gene expression (Figure 10). Angiogenesis and capillary sprouting as already seen on day 15, directly correlate with Type IV collagen expression as seen throughout the time period observations on day 15, 60, and 90 (Figure 10). Type IV collagen expression is also present on day 90, indicating the continuous angiogenic capillary sprouting as seen morphologically (Ripamonti et al., 1993).

A further word of caution on the clinical translation of the tissue engineering paradigm is needed as we have often stated in recent research output (Ripamonti et al., 2014). Tissue engineering and “*regenerative medicine in clinical contexts is on a different scale altogether when compared to animal models including non-human primate species that may or may not adequately translate and reproduce morphogen-related therapeutic responses in human patients*” (Ripamonti et al., 2006, 2007a, 2014). Perhaps no one else but David Williams (Williams, 2006) has so clearly questioned the tissue engineering paradigm in his manuscript that states that regenerative medicine and tissue engineering may “*prove to be a nadir out of which only success can emerge?* “ *or “is it a subject so fatally flawed by a misappropriation of medical principles and commercial hype that it can only serve to deceive and ultimately fail?*” (Williams, 2006).

The hype of tissue engineering and regenerative medicine took by surprise not only molecular and tissue biologists but also the more conservative clinician scientists, less inclined to espouse hyperbolic statements from both scientific *Journals* and the alerted media on the extraordinary discoveries of tissue biology (Williams, 2006; Cell Editorial, 2014a).

To be truth to regenerative medicine, the emergence of the tissue engineering dream and paradigm was indeed based on scientific discoveries that propelled molecular, tissue and developmental biology to previously unknown levels of knowledge (Williams, 2006; Cell Editorial, 2014a; Ripamonti et al., 2014). The often outstanding results in pre-clinical models including non-human primate species have invocated that procedures devised by the regenerative medicine' dream would provide capacity to regenerate tissues and organs at molecular and cellular levels, finally making spare parts for the human body (Ripamonti et al., 1997, 2008; Klar et al., 2014). This is not the case, however, and “*the possibility of abject failure is there for all to see*” (Williams, 2006).

The acid test of regenerative medicine is to translate in clinical contexts the often extraordinary results as shown in animal models, including non-human primate species (Ripamonti et al., 2014). As stated by Williams however “*none of the newly developed tissue engineering procedures are actually routinely used in clinical contexts*” (Williams, 2006). D. Williams, and 10 years after his Chapter contributed above, did state in his co-authored paper to *Biomaterials* (Tang et al., 2016) that “*skeletal tissue engineering has not yet achieved full translation into clinical practice as a consequence of several challenges*” (Tang et al., 2016). Together with the above more recent paper, it is worthwhile to also quote an additional paper by D. Williams in *Tissue Engineering* where it is stated that the problems of the clinical translation of tissue engineering are manifold, and that: “*At this stage, tissue engineers are simply unable to routinely use a tissue engineering approach to generate significant volumes of vascularized, innervated tissue*” (Williams, 2014).

The real challenge ahead is to critically re-visit the scientific performance of tissue engineering translational capacity in clinical contexts with the ultimate goal to humbly and serenely accept the lack of translational success and seek for answers going back to the board to mechanistically unravel what it is that went wrong with the unfulfilled promise of tissue engineering. A pertinent example is the still enthusiastic advocation of recombinant human bone morphogenetic proteins for therapeutic bone tissue engineering. The available uninspiring clinical trials data together with the evidence of life threatening side effects have indicated that this is now untenable (US Food and Drug Administration, 2008; Department of Justice, 2009; Williams et al., 2010; Carragee et al., 2011b; Centre for Devices and Radiological Health, 2011; Ripamonti et al., 2014, 2016).

The reconstruction of osseous defects in human patients is accomplished successfully with autogenous bone grafts. Whilst a lot is made of the morbidity of the harvesting of such grafts (by advocates of tissue engineering), massive craniomandibulofacial defects in humans can be successfully treated with autogenous bone grafts (Ripamonti et al., 2014; Ferretti et al., 2016). It is now mandatory to systematically identify the molecular and cellular bases responsible for the significant differences of various patterns of healing amongst mammals, and primates in particular. Could we possibly genetically analyze the primate-wound healing trait controlling the induction of tissue regeneration (McBrearty et al., 1998)? What is it that makes the human primate *Homo sapiens* heal with difficulties and uninspiringly when compared to animal models including non-human primates?

As we have previously stated “*Only a concerted genetic and molecular approach will break the boundary of super healing*” (Ripamonti, 2010). As Sanchez Alvarado and Yamanaka state, “*In biology and particularly in evolution, rules are meant to be broken…as most of evolutionary advances have arisen from the violation of pre-existing rules*” (Sanchez Alvarado and Yamanaka, 2014).

To end, is the widespread dream of deploying tissue engineering and regenerative procedures for the decaying human organs and tissues destined to simply remain a dream after several unfulfilled promises following, however, extraordinary results in pre-clinical animal models including non-human primates? Or “*Are we there yet?*” further stating that “*Tissue engineering and stem cell industry has stabilized and is on a path pointing toward continued success*.” (Jaklenec et al., 2012). In a more recent Editorial in *Nature Regenerative Medicine* discussing again “*Are we there yet?*” Stephen Badylak and Nadia Rosenthal state however: “*For all the progress we have made in the fast moving field of regenerative medicine, the capacity to routinely restore functional tissue following traumatic injury or degenerative disease is still beyond reach*” (Badylak and Rosenthal, 2017).

Is there a way ahead? Our research work has invocated that the only way ahead is to finally study molecularly and genetically the genome of the genus Papio vs. the genome of the genus Homo to try to unravel why as *Homo sapiens* we do not heal let alone regenerate as *Papio ursinus* does. This novel research avenue may help to mechanistically resolve the regenerative capacities of primate' tissues including humans, possibly unraveling the fundamental mechanisms of unique human biology (Cell Editorial, 2014b).

## Author contributions

This review is sole authored by UR that initiated research on the described coral-derived macroporous constructs, studied the induction of bone formation by the concavities of the substratum, initiated further research, raised grants and published several data including US and European patents as inventor, i.e., UR.

### Conflict of interest statement

The author declares that the research was conducted in the absence of any commercial or financial relationships that could be construed as a potential conflict of interest. The reviewers VD and VT and handling Editor declared their shared affiliation.
